# Association of COVID-19 Government-Instituted Mask Mandates With Incidence of Mask Use Among Children in Alberta, Canada

**DOI:** 10.1001/jamanetworkopen.2023.17358

**Published:** 2023-06-08

**Authors:** Lyndsey M. Hahn, Emilie Manny, Gurvinder Dhaliwal, Joyce Chikuma, Joan Robinson, Wendy Lou, Padmaja Subbarao, Stuart E. Turvey, Elinor Simons, Rhonda C. Bell, Nicole Letourneau, Carmen Charlton, Piush J. Mandhane

**Affiliations:** 1 Department of Pediatrics, Faculty of Medicine & Dentistry, University of Alberta, Edmonton, Alberta, Canada; 2 Women and Children’s Health Research Institute, Edmonton, Alberta, Canada; 3 Dalla Lana School of Public Health, University of Toronto, Toronto, Ontario, Canada; 4 Department of Pediatrics, Hospital for Sick Children, University of Toronto, Toronto, Ontario, Canada; 5 Department of Pediatrics, BC Children’s Hospital, University of British Columbia, Vancouver, British Columbia, Canada; 6 Department of Pediatrics & Child Health, Children’s Hospital Research Institute of Manitoba, University of Manitoba, Winnipeg, Manitoba, Canada; 7 Department of Agricultural, Food and Nutritional Science, Faculty of Agricultural, Life and Environmental Sciences, University of Alberta, Edmonton, Alberta, Canada; 8 Faculties of Nursing & Cumming School of Medicine (Pediatrics Psychiatry & Community Health Sciences), University of Calgary, Calgary, Alberta, Canada; 9 Department of Laboratory Medicine and Pathology, University of Alberta, Edmonton, Alberta, Canada; 10 Public Health Laboratory, Alberta Precision Laboratories, Edmonton, Alberta, Canada; 11 Li Ka Shing Institute of Virology, University of Alberta, Edmonton, Alberta, Canada

## Abstract

**Importance:**

To help prevent the spread of SARS-CoV-2, government-instituted nonpharmaceutical interventions (eg, social distancing, mask use, isolating), a provincewide government-instituted mask mandate occurred on December 8, 2020, in Alberta, Canada, although some local jurisdictions implemented an earlier mask mandate. There remains a limited understanding of the association between government-implemented public health measures and individual health behaviors of children.

**Objective:**

To examine the association between government mask mandates and mask use among children in Alberta, Canada.

**Design, Setting, and Participants:**

A cohort of children from Alberta, Canada, was recruited to examine longitudinal SARS-CoV-2 serologic factors. Parents were prospectively asked about their child’s mask use in public places every 3 months (5-point Likert scale: never to always) from August 14, 2020, to June 24, 2022. A multivariable logistic generalized estimating equation was used to examine government mandatory masking mandates and child mask use. Child mask use was operationalized into a single composite dichotomous outcome by grouping parents who reported their child often or always wore a mask vs those who reported their child never, rarely, or occasionally wore a mask.

**Exposures:**

The primary exposure variable was the government masking mandate (began on different dates in 2020). The secondary exposure variable was government private indoor and outdoor gathering restrictions.

**Main Outcomes and Measures:**

The primary outcome was parent report of child mask use.

**Results:**

A total of 939 children participated (467 female [49.7%]; mean [SD] age, 10.61 [1.6] years). The odds of parents’ report of child mask use (often or always) was 18.3 times higher (95% CI, 5.7-58.6; *P* < .001; risk ratio, 1.7; 95% CI, 1.5-1.8; *P* < .001) with the mask mandate on compared with the mask mandate off. There was no significant change in mask use over the course of the mask mandate due to time. In contrast, each day with the mask mandate off was associated with a 1.6% decrease in mask use (odds ratio, 0.98; 95% CI, 0.98-0.99; *P* < .001).

**Conclusions and Relevance:**

The results of this study suggest that government-mandated mask use and providing the public with up-to-date health information (eg, case counts) is associated with increased parent-reported child mask use, while increasing time without a mask mandate is associated with decreased mask use.

## Key Points

**Question:**

Are government mask mandates associated with mask use among children in Alberta, Canada?

**Findings:**

This cohort study of 939 children (age, 8-13 years) from August 2020 to June 2022 examined government masking mandates and child mask use; the odds of parents’ report of child mask use (often or always) was 18.3 times higher during the mask mandate compared with when there was no mandate, which was a significant difference. Each day without the mask mandate was associated with a 1.6% decrease in mask use.

**Meaning:**

The findings of this study suggest the importance of public health messaging and time since the mask mandate was lifted.

## Teaser

This cohort study examines parent-reported incidence of masking among children during periods of the COVID-19 pandemic with vs without government mask mandates in Alberta, Canada.

## Introduction

The World Health Organization declared a global pandemic in relation to COVID-19 outbreaks on March 11, 2020.^1^ Governments instituted nonpharmaceutical interventions (NPIs) (eg, social distancing, mask use, and isolating) to help prevent the spread of SARS-CoV-2 infection.^2,3^ Child masking was associated with fewer closures of childcare programs in the US during the COVID-19 pandemic.^4^

There remains a limited understanding of the association between government-implemented public health measures and individual health behaviors of children. A Canadian study^5^ found that women and older participants were more likely to perceive public health measures as effective in lowering the transmission of COVID-19. Data from France (December 2020)^6^ reveal that most parents (95.4%) adhered to mask use in schools for children aged 6 years and older as mask use was mandatory (ie, within schools and indoor public places). However, a large proportion of the parents disagreed (63.3%) with the mandatory mask mandate and 54.6% of the parents did not understand the reasons behind mask recommendations. We could not find any studies that examined the association between government-mandated public health measures and NPI use among children. Research on social distancing behavior during the COVID-19 pandemic suggests that government policy was associated with increased NPI (ie, social distancing and staying home) behavior for adults.^7^

We examined the association between government mask mandates and parent-reported mask use (primary outcome) and avoiding crowded places/gatherings (secondary outcome) among children aged 8 to 13 years, using data from a longitudinal pediatric SARS-CoV-2 serologic cohort. We hypothesized that government-mandated public health measures would be associated with an increase in parent-reported NPI use among children.

## Methods

### Study Participants

Parents and their children aged 8 to 13 years were recruited to a study examining longitudinal SARS-CoV-2 serologic characteristics (Alberta pediatric serologic cohort) from August 14, 2020, through March 12, 2021. Participants were recruited from the CHILD Cohort Study (Edmonton site), the Alberta Pregnancy Outcomes and Nutrition Study, and free media (eg, social media, earned media). Study invitations were extended to participants’ family and friends. Only 1 child per household was recruited. Written consent was obtained from study participants. Written informed consent was provided by parents and assent by children, and participants received financial compensation. The University of Alberta Research Ethics Board approved the Alberta COVID-19 Serology Study and the Alberta COVID-19 Serology Study: Secondary Data Analysis. This cohort study follows the Strengthening the Reporting of Observational Studies in Epidemiology (STROBE) reporting guideline.

### Study Variables

For the primary outcome of mask use, parents were prospectively (quarterly questionnaires, every 3 months) asked about their child’s mask use in public places for the previous 3 months (5-point Likert scale: never [0], rarely [1], occasionally [2], often [3], or always [4]) from August 14, 2020, to June 24, 2022.

The primary exposure variable was government-mandated masking. Mandatory masking within indoor public locations was first implemented on different dates depending on location within the province of Alberta (eTable 1 in Supplement 1). The earliest mask mandates occurred on August 1, 2020, in Edmonton and Calgary. A provincial mask mandate was implemented on December 8, 2020.

**Figure 1.**
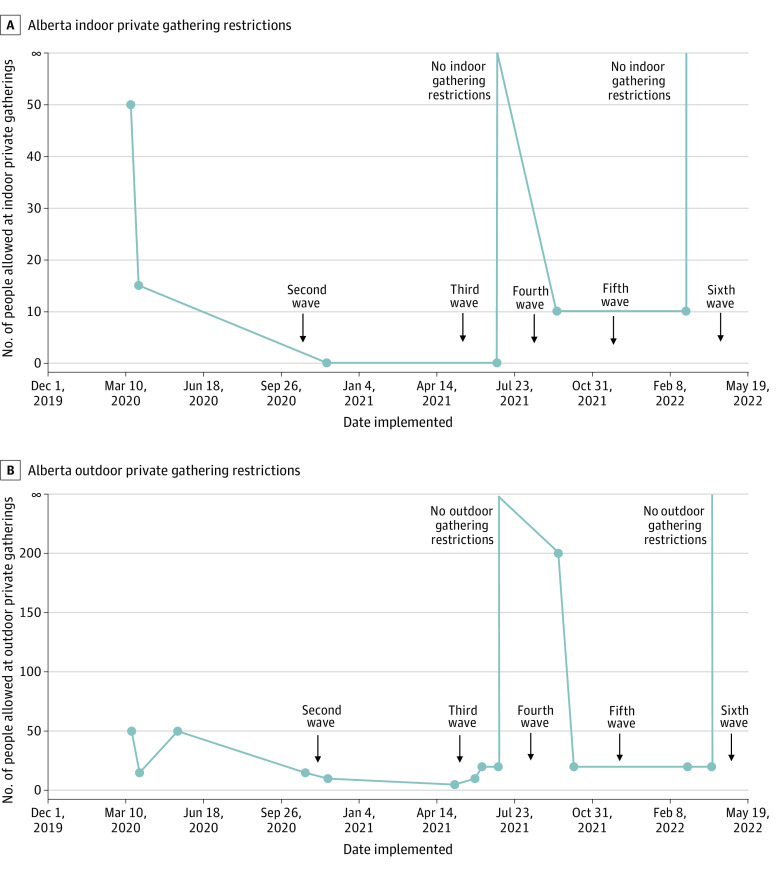
Private Gathering Restrictions A, Alberta indoor private gathering restrictions. B, Alberta outdoor private gathering restrictions.

The secondary exposure variable was government restrictions on private indoor and outdoor gatherings. The Alberta Chief Medical Officer of Health implemented various provincewide private indoor (Figure 1A) and outdoor (Figure 1B) gathering restrictions throughout the COVID-19 pandemic (eTable 2 and eTable 3 in Supplement 1).

Potential confounding variables considered in the analysis included Alberta COVID-19 active case rate per 100 000 in the participant’s area,^8^ parent occupation, parent educational level, family income, location of residence (urban vs rural), and time within a mask mandate state. Active case count was not available on the Government of Alberta website after May 2022. Additional NPI use assessed at the same time as mask use, using the same Likert scale, included (1) avoided crowded places or gatherings (secondary outcome), (2) practiced physical distancing in public places, (3) avoided common greetings (eg, handshakes and hugs), (4) limited contact with people at higher risk (eg, older relative), and (5) quarantined because the child may have been exposed to SARS-CoV-2, but did not show symptoms. Parents reported their willingness to receive a COVID-19 vaccination themselves throughout the study period (ie, Would you be willing to take a vaccine if/when one becomes available? Is a vaccine to COVID-19 available to you now? Have you been vaccinated against COVID-19?).

### Statistical Analysis

Analysis was completed on data obtained up to June 30, 2022. We excluded children with a SARS-CoV-2 infection before recruitment confirmed by reverse transcription polymerase chain reaction testing. Child mask use and avoiding crowded places or gatherings were analyzed using a dichotomous variable (ie, never or rarely or occasionally vs often or always). A multivariable logistic generalized estimating equation (GEE), controlling for clustering within individuals, was used to examine associations between government restrictions (primary exposure variable) and child mask use (primary outcome) and avoiding crowded places or gatherings (secondary outcome). Robust SEs were used with the GEE analyses. Odds ratios (ORs) may provide a more extreme result when outcomes are not rare. Therefore, risk ratios (RRs) were also computed to analyze the data^9^ and were computed using Stata, version 17.0 (StataCorp LLC) margins (personal communication, Russell Localio, PhD, March 15, 2023). A missing data category was analyzed for all predictor categorical variables (eTable 4 and eTable 5 in Supplement 1). Government-mandated masking (primary exposure variable) was considered a time-dependent variable. Active case count per 100 000 in the participant’s area was normalized (unity-based scaling from 0 to 1) based on the maximum and minimum COVID-19 case counts reported to that date. A mean replace method was used to handle missing data for active case count.

We hypothesized that longitudinal mask use would be clustered within an individual (eg, often or always wear a mask even with mandates off vs rule followers). A secondary analysis used group-based trajectory modeling (traj package in Stata) to identify longitudinal trajectories in child mask use and avoiding crowded places or gatherings over time.^10,11^ Multinomial logistic regression was then used to identify predictors of an individual being in a trajectory (the probability of being in a trajectory). All a priori hypotheses were 2-sided, with findings significant at *P* ≤ .05. Statistical analyses were completed using Stata, version 17.0.^12^

## Results

There were 1670 families identified as interested in the study. After eligibility criteria were met, 939 participants (467 [49.7%] female, 461 [49.1%] male) were included in the analysis (Table). eFigure 1 in Supplement 1 shows participant recruitment and sample size. eFigure 2 in Supplement 1 shows participant response throughout the study. The mean (SD) age of the children was 10.61 (1.6) years. Mean household income was CAD $112 524 (approximately US $83 830), with 63.0% of parents having completed a university-level bachelor’s or graduate degree (eg, master’s or doctorate). More than 27% of parents worked in a hospital or other health care setting and 65% of the families lived in Edmonton or Calgary (major urban centers). We found no statistically significant differences between participant demographic characteristics who remained in the study compared with those who withdrew from the study (eTable 6 in Supplement 1).

**Table.** Study Demographic Characteristics in 939 Participants

Characteristic	Value
**Child**	
Age, mean (SD), y	10.6 (1.6)
Sex at birth, No. (%)	
Female	467 (49.7)
Male	461 (49.1)
Missing or other	11 (1.2)
**Parent/household**	
Household income based on postal code, mean (SD), $CAD	112 524.2 (27 082.28)
Household income missing, No. (%)	17 (1.8)
Home location, No. (%)	
Urban (ie, Edmonton or Calgary)	611 (65.1)
Rural	324 (34.5)
Missing	4 (0.43)
Education (highest level completed), No. (%)	
High school graduation or less	63 (6.7)
Trade certificate, vocational school, or apprenticeship training	49 (5.2)
Nonuniversity certificate or diploma from a community college, CEGEP	226 (24.1)
University bachelor’s degree	435 (46.3)
University graduate degree (eg, master’s or doctorate)	157 (16.7)
Prefer not to answer	9 (0.96)
Occupation since March 1, 2020	
Hospital or health care facility worker	255 (27.2)
First responder (paramedic, firefighter, police officer)	42 (4.5)
Childcare worker	23 (2.4)
Teacher	102 (10.9)
Other	517 (55.1)

Abbreviation: CEGEP, collège d'enseignement général et professionnel.

### Child Mask Use

**Figure 2.**
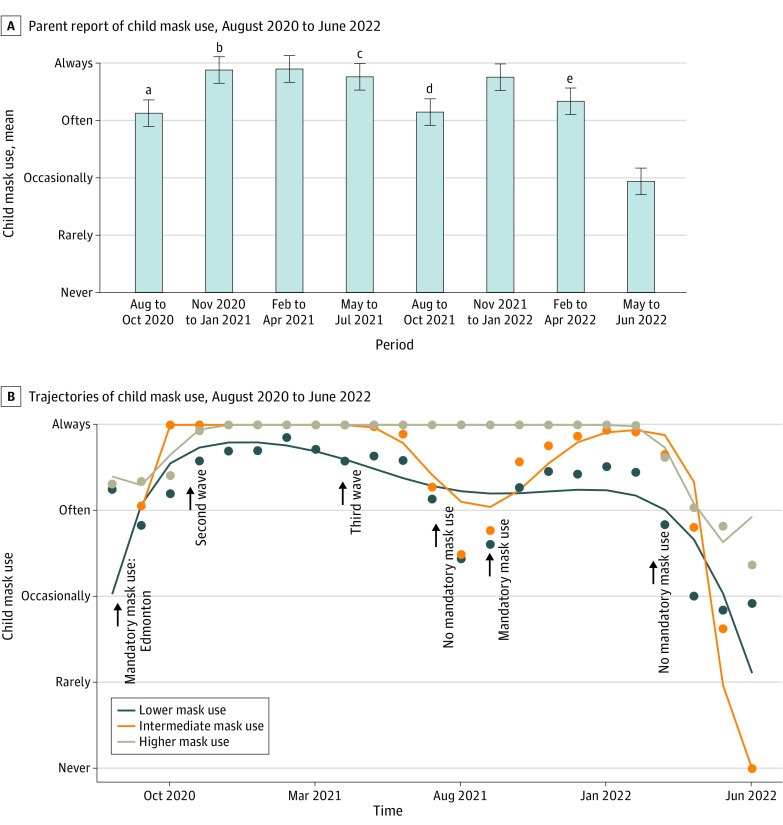
Child Mask Use A, Parent report of child mask use from August 2020 to June 2022. B, Trajectories of child mask use from August 2020 to June 2022. ^a^Mandatory use in Edmonton, August 1. ^b^Mandatory use in Alberta, December 8. ^c^No mandatory use in Alberta, June 1. ^d^Mandatory use in Alberta, September 4. ^e^No mandatory use in Alberta, March 1.

Child mask use increased from a mean (SD) of 3.1 (0.69) (4 is the maximum mask use response [always]) from August 14 through October 31, 2020, to 3.9 (0.43) from November 1, 2020, through January 31, 2021 (Figure 2A). Parents reported a mean (SD) decrease in reported child mask use of 3.1 (0.93) from August 1 to October 31, 2021. Mask use was no longer mandatory for indoor public places from July 1, 2021. Mandatory mask use for indoor public places was reimplemented on September 4, 2021. Reported mask use was higher during the November 2021 to January 2022 time frame (mean [SD] response, 3.8 [0.50]). Mask use was again lower from February 1 to April 30, 2022 (mean [SD] response, 3.3 [0.86]), and from May 1 to June 30, 2022 (mean [SD] response 1.9 [1.1]), when the mandatory mask mandate was lifted on March 1, 2022. eFigure 3 in Supplement 1 provides the distribution of child mask use (ie, never, rarely, occasionally, often, or always) at each time point.

#### GEE Analysis

**Figure 3.**
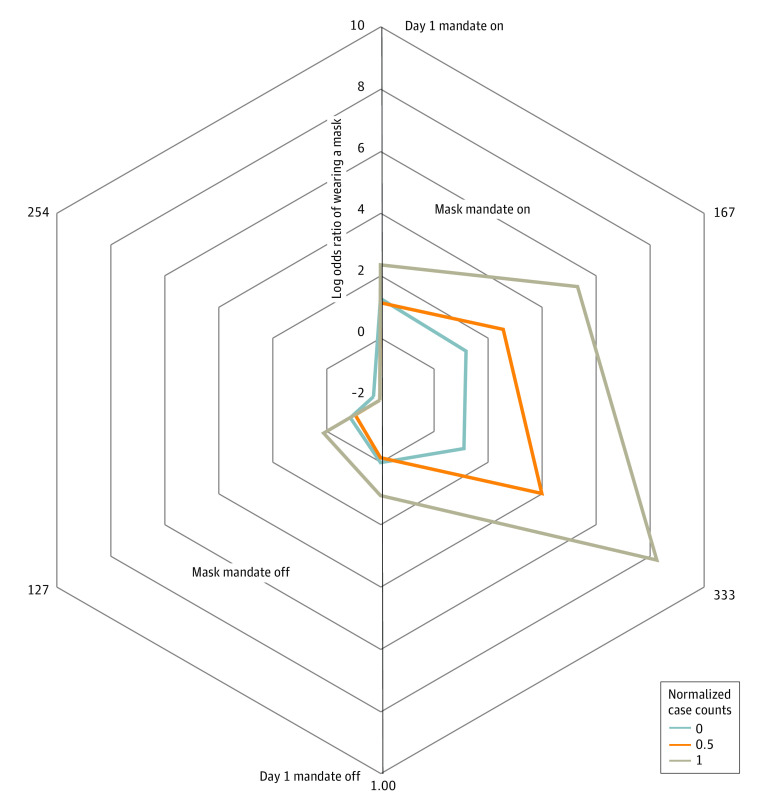
Child Mask Use by Time (Days) With and Without a Mask Mandate by Normalized Case Counts

The odds of parents’ report of child mask use (often or always) was 18.3 (95% CI, 5.7-58.6; *P* < .001) times higher when the mask mandate was on compared with when the mask mandate was off (Figure 3; eTable 4 in Supplement 1). There was no significant change in mask use over the course of the mask mandate. In contrast, each day with the mask mandate off was associated with a 1.6% decrease in mask use (OR, 0.98; 95% CI, 0.98-0.99; *P* < .001). Increasing mask use was associated with the square of the normalized case count; maximum normalized case counts were associated with an 11-fold increase in mask use (OR, 11.4; 95% CI, 1.8-72.3; *P* < .05). The odds of parents reporting that their child often or always used a mask was 5.4 (95% CI, 4.2-7.0; *P* < .001) times higher when parents reported that their child often or always avoided crowded places or gatherings. The odds of parents reporting that their child often or always used a mask was 0.49 (95% CI, 0.29-0.81; *P* < .01) times lower when parents reported that they were not willing to receive a COVID-19 vaccination and currently not vaccinated. No associations were found between child mask use and household income. In a sensitivity analysis, combining the missing groups into the largest category did not result in a significant change in the direction or magnitude of results (eTable 7 in Supplement 1).

Risk ratio analysis showed that the risk of the parents’ report of child mask use often or always was 1.7 (95% CI, 1.5-1.8; *P* < .001) times higher when the mask mandate was on compared with when the mask mandate was off (eTable 4 in Supplement 1). The risk of the parents’ report of their child using a mask often or always was 1.14 (95% CI, 1.11-1.17; *P* < .001) times higher when parents also reported that their child often or always avoided crowded places or gatherings. There was a 4% reduction (RR, 0.96; 95% CI, 0.93-1.00; *P* < .05) in the risk of parents’ reporting that their child often or always used a mask when parents reported that they were not willing to receive a COVID-19 vaccination and were not currently vaccinated.

#### Trajectory Analysis

We identified 3 mask use trajectories from August 2020 to June 2022 (Figure 2B): lower mask use (34.7% of participants), intermediate mask use (34.9%), and higher mask use (30.5%). Parents who were willing to receive a COVID-19 vaccination were 2.0 times more likely to be in the higher mask use group compared with the intermediate mask use group (OR, 2.0; 95% CI, 1.4-2.9; *P* < .001) and 45% less likely to be in the lower mask use group (OR, 0.55; 95% CI, 0.40-0.75; *P* < .001) (eTable 8 in Supplement 1). Participants who lived in Edmonton or Calgary were 1.8 (OR, 1.8; 95% CI, 1.6-2.0; *P* < .001) times more likely to be in the higher mask use group compared with the intermediate mask use group. Participants in the higher mask use group were 50% less likely to have a household income in the 4th quartile compared with the intermediate mask use group (OR, 0.50; 95% CI, 0.47-0.54; *P* < .001). No associations were found between groups for parent educational level or for parents who worked in a hospital or other health care facility.

### Children Avoiding Crowded Places or Gatherings

**Figure 4.**
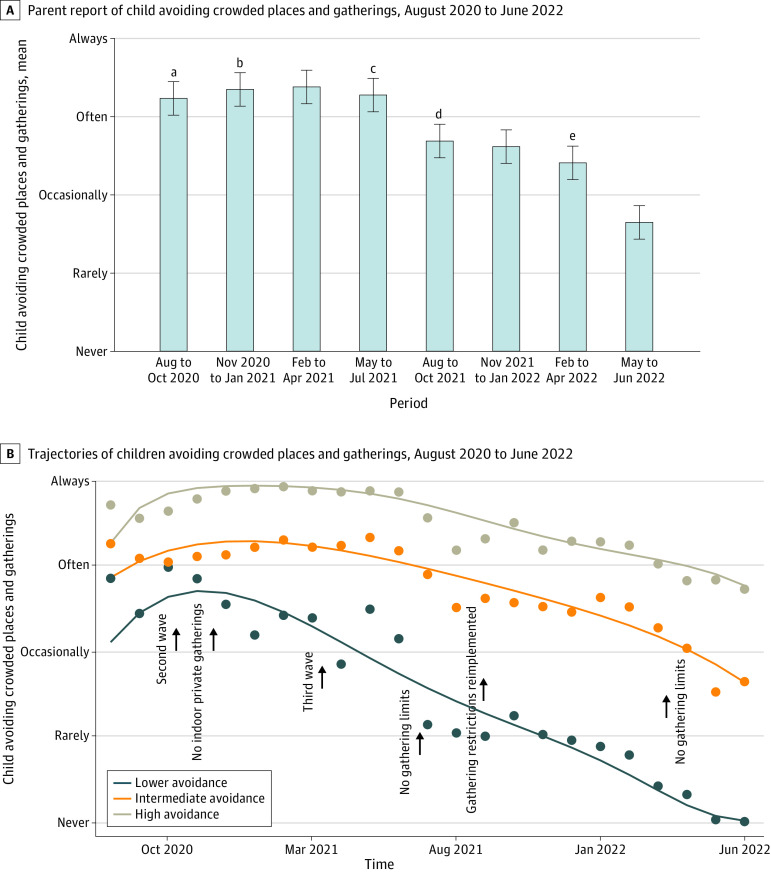
Avoidance of Crowded Places and Gatherings A, Parent report of child avoiding crowded places and gatherings from August 2020 to June 2022. B, Trajectories of children avoiding crowded places and gatherings from August 2020 to June 2022. ^a^First gathering restrictions, March 17. ^b^No indoor private gatherings, November 24. ^c^No gathering limits, July 1. ^d^Indoor gathering restrictions, September 16. ^e^No gathering limits, March 1.

Mean (SD) response for children avoiding crowded places or gatherings from August to October 2020 was 3.2 (0.63) (4 is the maximum avoiding response; always) (Figure 4A). The first gathering restrictions were introduced in Alberta on March 17, 2020, with no indoor private gatherings allowed starting November 24, 2020. Children consistently often or always avoided gatherings from November 2020 to January 2021 (3.4 [0.64]) and February to April 2021 (3.4 [0.72]). Gathering limits were removed July 1, 2021, but were reintroduced September 16, 2021. Children avoided crowded places or gatherings less often from August to October 2021 (2.7 [0.92]). Children continued to avoid crowded places or gatherings less often through to June 2022 (May-June 2022, 1.6 [1.2]).

#### GEE Analysis

There were significant differences between government private gathering rules and normalized active case counts on parents’ report of children avoiding crowded places or gatherings. The odds of parents’ report of children avoiding crowded places or gatherings often or always was 2.0 (95% CI, 1.6-2.6; *P* < .001) times higher when no private gatherings were allowed by the government (eTable 5 in Supplement 1). The odds of parents’ report of children avoiding crowded places or gatherings often or always was 1.4 (95% CI, 1.1-1.9; *P* < .05) times higher when normalized active case counts were taken into account. The odds of parents’ report of children avoiding crowded places or gatherings often or always was 0.66 (95% CI, 0.48-0.93; *P* < .05) times lower when parents reported that they were not willing to receive a COVID-19 vaccination and were not vaccinated against COVID-19. The odds of parents’ report of children avoiding crowded places or gatherings often or always was 0.56 (95% CI, 0.44-0.71; *P* < .001) times lower when parents reported that they lived outside of Edmonton or Calgary. No association was found between reports of children avoiding crowded places or gatherings often or always and the mandatory mask mandate. Furthermore, no association was found between reports of children avoiding crowded places or gatherings and household income. In a sensitivity analysis, combining the missing groups into the largest category did not result in a significant change in the direction or magnitude of the results (eTable 9 in Supplement 1).

The risk of parents’ report of children avoiding crowded places or gatherings often or always was 1.1 (95% CI, 1.1-1.2; *P* < .001) times higher when no private gatherings were allowed by the government (eTable 5 in Supplement 1). There was a 10% reduction of risk (RR, 0.90; 95% CI, 0.85-0.94; *P* < .001) of parents’ report of children avoiding crowded places or gatherings often or always when parents reported that they lived outside of Edmonton or Calgary.

#### Trajectory Analysis

We identified 3 trajectories of children avoiding crowded places or gatherings (Figure 4B): lower avoidance (9.7% of participants), intermediate avoidance (57.4), and higher avoidance (33.0%). Parents who reported being more likely to be willing to receive a COVID-19 vaccination were 86% less likely (OR, 0.14; 95% CI, 0.09-0.02; *P* < .001) to be in the lower avoidance group vs the intermediate group (eTable 8 in Supplement 1). Participants who lived outside of Edmonton or Calgary (more rural) were 2.2 (95% CI, 1.8-2.6; *P* < .001) times more likely to be in the lower avoidance group vs the intermediate group. In contrast, participants who lived in Edmonton or Calgary (major urban centers) were 1.4 (95% CI, 1.3-1.6; *P* < .001) times more likely to be in the higher avoidance group vs the intermediate group. Parents in the higher avoidance group were less likely to work in a hospital or another health care setting compared with the intermediate group (OR, 0.70; 95% CI, 0.66-0.74; *P* < .001).

## Discussion

Using data from the Alberta pediatric SARS-CoV-2 serologic cohort, we found that both individual and family and societal factors, as well as government rules, were associated with NPI use in children. Child mask use was higher with higher community SARS-CoV-2 case counts, suggesting that public reporting of case counts (eg, waves and spikes) has value to motivate the public. Individual and family factors, such as willingness to receive the SARS-CoV-2 vaccine and urban vs rural location, were also associated with NPI. The longer the time with the mask mandates off, the lower the odds of parents reporting their child wearing a mask. These results suggest that NPI use is not homogeneous and tailored messaging is required for behavior and location. Our findings emphasize the importance of public health messaging and time out of a mandate state on NPI use in children.

We could not identify any previous research examining the association between government-mandated public health measures and NPI use among children. Previous research has focused on the association of individual and family factors, such as perceived risk, susceptibility to illness, or understanding of COVID-19,^2,5,13,14^ and NPI use. Data from Canada and the US have shown that child mask use is highly influenced by parent intention for their child to wear a mask independent of whether parents hold a positive attitude about mask wearing or show efficacy in teaching children to wear masks.^15^ Similarly, we found parent willingness to receive the SARS-CoV-2 vaccine was associated with both NPI outcomes in their child. This supports current findings in the literature suggesting an association between COVID-19 vaccination status and participation in NPIs.^16^ Additional research is needed to further understand how other individual and family factors may influence NPI use in children. Our finding that child mask use was higher with higher community SARS-CoV-2 case counts, suggesting that public reporting of case counts has value is consistent with other research highlighting the importance of testing and communication during a pandemic.^7^

Further research on integrating individual, family, and societal factors with NPI use among children and families across jurisdictions may help elucidate the generalizability of our findings. A national study that includes samples from multiple provinces would increase sample size and allow for a more in-depth analysis of variations in public health policy. Additional work will also examine the association between NPI use and child’s risk for SARS-CoV-2 infection.

### Strengths and Limitations

A strength of this study was the use of prospective questionnaires that assessed NPI use throughout the COVID-19 pandemic from August 2020 onward. This study was able to assess multiple potential confounders (ie, Alberta COVID-19 active case rate in the participant’s area, parent willingness to receive a COVID-19 vaccination, parent occupation, parent educational level, family income, and location of residence). Furthermore, this study used longitudinal data with more than a 1-year follow-up period allowing us to examine changes in government policy and their association with NPI use.

This study has limitations. The limited sample size within individual communities and quarterly questionnaires did not allow us to account for more nuanced changes in government public health measures through time. For example, although mandatory masking for indoor public places was reimplemented in September 2021, individuals could participate in indoor dining at restaurants without a mask with members outside of their household if all individuals were fully vaccinated. Previous restrictions were limited to indoor dining at the same table only if each person was living within the same household. In addition, the location (indoor or outdoor) of gatherings was not determined and children were not asked directly about their behavior, attitudes, or beliefs. Future studies that include child reports may reduce responder bias.

Differences between school boards for their NPI mandates could not be fully accounted for in the analysis. The government implemented a mandatory masking mandate for schools in September 2021; however, many school boards implemented their own guidelines for indoor school activities.^17,18,19^ Subsequently, on February 14, 2022, the Government of Alberta removed mandatory masking for children younger than 13 years and students of any age attending kindergarten through grade 12 in school activities (across all school boards). This study also had limited generalizability of results due to the restricted geographic nature of the sample.

## Conclusion

Using data from the Alberta pediatric SARS-CoV-2 serologic cohort, we found that both individual and family and societal factors were associated with NPI use in children. Our findings emphasize the importance of understanding how time and public health messaging may influence NPI use. Use of NPIs is not homogeneous and tailored messaging is required for each behavior and location. This research may be the starting point to inform the effectiveness of implementing government mandated public health measures within the ongoing pandemic and future pandemics.

## Notes

Supplement 1. **eTable 1.** Mandatory Masking in Alberta **eTable 2.** Alberta Indoor Private Gathering Restrictions and CMOH Orders **eTable 3.** Alberta Outdoor Private Gathering Restrictions and CMOH Orders **eTable 4.** Child Mask Use (Often or Always): Supplementary Analysis **eTable 5.** Child Avoiding Crowded Places/Gatherings (Often or Always): Supplementary Analysis **eTable 6.** A Comparison of Demographics for Participants Who Withdrew From the Study and Participants Who Stayed in the Study **eTable 7.** Child Mask Use (Often or Always) With Missing Data Incorporated Into the Largest Category **eTable 8.** Child Mask Use and Avoiding Crowded Places/Gatherings: Trajectory Group Analysis **eTable 9.** Child Avoiding Crowded Places/Gatherings: (Often or Always) With Missing Data Incorporated Into the Largest Category **eFigure 1.** Participant Recruitment and Sample Size **eFigure 2.** Participant Response Throughout the Study **eFigure 3.** Child Mask Use (Percentage)

Supplement 2.
Data Sharing Statement
