# Supplementary material for: Association of COVID-19 Government-Instituted Mask Mandates With Incidence of Mask Use Among Children in Alberta, Canada
Source: JAMA Netw Open. 2023 Jun 8;6(6):e2317358. doi: 10.1001/jamanetworkopen.2023.17358 (PMC10251214; doi:10.1001/jamanetworkopen.2023.17358)
Supplement: Supplement 1. — eTable 1. Mandatory Masking in Alberta eTable 2. Alberta Indoor Private Gathering Restrictions and CMOH Orders eTable 3. Alberta Outdoor Private Gathering Restrictions and CMOH Orders eTable 4. Child Mask Use (Often or Always): Supplementary Analysis eTable 5. Child Avoiding Crowded Places/Gatherings (Often or Always): Supplementary Analysis eTable 6. A Comparison of Demographics for Participants Who Withdrew From the Study and Participants Who Stayed in the Study eTable 7. Child Mask Use (Often or Always) With Missing Data Incorporated Into the Largest Category eTable 8. Child Mask Use and Avoiding Crowded Places/Gatherings: Trajectory Group Analysis eTable 9. Child Avoiding Crowded Places/Gatherings: (Often or Always) With Missing Data Incorporated Into the Largest Category eFigure 1. Participant Recruitment and Sample Size eFigure 2. Participant Response Throughout the Study eFigure 3. Child Mask Use (Percentage) [file jamanetwopen-e2317358-s001.pdf]

Supplementary Online Content

Hahn LM, Manny E, Dhaliwal G, et al. Association of COVID-19 government-instituted mask mandates with incidence of mask use among children in Alberta, Canada. *JAMA Netw Open*. 2023;6(6):e2317358. doi:10.1001/jamanetworkopen.2023.17358

eTable 1. Mandatory Masking in Alberta

eTable 2. Alberta Indoor Private Gathering Restrictions and CMOH Orders

eTable 3. Alberta Outdoor Private Gathering Restrictions and CMOH Orders

eTable 4. Child Mask Use (Often or Always): Supplementary Analysis

eTable 5. Child Avoiding Crowded Places/Gatherings (Often or Always): Supplementary Analysis

eTable 6. A Comparison of Demographics for Participants Who Withdrew From the Study and Participants Who Stayed in the Study

eTable 7. Child Mask Use (Often or Always) With Missing Data Incorporated Into the Largest Category

eTable 8. Child Mask Use and Avoiding Crowded Places/Gatherings: Trajectory Group Analysis

eTable 9. Child Avoiding Crowded Places/Gatherings: (Often or Always) With Missing Data Incorporated Into the Largest Category

eFigure 1. Participant Recruitment and Sample Size

eFigure 2. Participant Response Throughout the Study

eFigure 3. Child Mask Use (Percentage)

This supplementary material has been provided by the authors to give readers additional information about their work.

© 2023 Hahn LM et al. *JAMA Network Open.*

eTable 1: Mandatory masking in Alberta

Mandatory masking all indoor public places: Date implemented	Location*
August 1, 2020	Edmonton
August 1, 2020	Calgary
August 8, 2020	St. Albert
August 10, 2020	Beaumont
August 20, 2020	Sturgeon County
October 5, 2020	Sherwood Park
October 5, 2020	Ardrossan
October 8, 2020	Leduc/Leduc County
October 14, 2020	Fort Saskatchewan
October 23, 2020	Spruce Grove
October 27, 2020	Stony Plain
October 27, 2020	County of Barrhead
October 28, 2020	Westlock
November 3, 2020	Wetaskiwin
November 3, 2020	Whitecourt
November 12, 2020	Parkland County
November 12, 2020	Elk Point
November 13, 2020	Morinville
November 22, 2020	Athabasca
November 23, 2020	Devon
November 24, 2020	Tofield
November 25, 2020	Beaver County
November 27, 2020	Gibbons
November 30, 2020	Vegreville
December 8, 2020	Lacombe
December 8, 2020	Radway
December 8, 2020	Bruderheim
December 8, 2020	Innisfail
December 8, 2020	Wainwright

*Locations shown represent locations participants in study reside

© 2023 Hahn LM et al. *JAMA Network Open.*

eTable 2 Alberta indoor private gathering restrictions and CMOH Orders

**CMOH Order**	**Alberta indoor private gathering restriction (Date implemented)**
CMOH Order 02-2020	Limit of 50 people (March 17, 2020)
CMOH Order 07-2020	Limit of 15 people (March 27, 2020)
CMOH Order 38-2020	No indoor private gatherings permitted (November 24, 2021)
	No restrictions for indoor private gatherings (July 1, 2021)
CMOH Order 44-2021	Limit of 10 (2 households) for vaccinated (vaccine eligible) people. No indoor private gatherings for unvaccinated people (September 16, 2021)
CMOH Order 09-2022	Rescinds CMOH Order 08-2022 (Limit of 10 people) (February 8, 2022). No restrictions for indoor private gatherings (February 29, 11:59pm)

© 2023 Hahn LM et al. *JAMA Network Open.*

eTable 3 Alberta outdoor private gathering restrictions and CMOH Orders

CMOH Order	Alberta outdoor gathering restriction (Date implemented)
CMOH Order 02-2020	Limit of 50 people (March 17, 2020)
CMOH Order 07-2020	Limit of 15 people (March 27, 2020)
CMOH Order 20-2020	Limit of 50 people (May 15, 2020)
CMOH Order 35-2020	Limit of 15 people (October 25, 2020)
CMOH Order 38-2020	Limit of 10 people (November 24, 2020)
CMOH Order 19-2021	Limit of 5 people (May 6, 2021)
CMOH Order 30-2021	Limit of 10 people (June 1, 2021)
CMOH Order 31-2021	Limit of 20 people (June 10, 2021)
	No outdoor gathering restrictions (July 1, 2021)
CMOH Order 42-2021	Limit of 200 people (September 16, 2021)
CMOH Order 47-2021	Limit of 20 people (October 6, 2021)
CMOH Order 09-2022	Rescinds CMOH Order 08-2022. No outdoor gathering restrictions (February 29, 11:59pm).

© 2023 Hahn LM et al. *JAMA Network Open.*

eTable 4: Child mask use (often or always): Supplementary analysis

	Child mask use (parent report)			
	Odds ratio (95%CI)	p-value	Risk ratio (95% CI)	p-value
Government mandatory masking	18.3 (5.7, 58.6)	0.000	1.7 (1.5, 1.8)	0.000
Parent vaccine: not willing to receive and not vaccinated	0.49 (0.29, 0.81)	0.006	0.96 (0.93, 1.0)	0.03
Parent vaccine: not available and willing to receive	1.2 (0.52, 2.8)	0.66	1.01 (0.97, 1.06)	0.56
Parent vaccine: missing	1.8 (0.94, 3.5)	0.08	1.04 (1.00, 1.07)	0.03
Avoided crowded places/gatherings (often or always)	5.4 (4.2, 7.0)	0.000	1.14 (1.11, 1.17)	0.000
Avoided crowded places/gatherings missing	0.35 (0.08, 1.5)	0.15	0.79 (0.63, 0.99)	0.04
Alberta COVID-19 active case rate per 100,000 (normalized)	0.02 (0.05, 0.1)	0.000	N/A	N/A
Alberta COVID-19 active case rate per 100,000 (normalized) # Time in state	1.00 (0.99, 1.01)	0.80	N/A	N/A
Government mandatory masking # Alberta COVID-19 active case rate per 100,000 (normalized)	0.0005 (0.00003, 0.009)	0.000	N/A	N/A
Alberta COVID-19 active case rate per 100,000 (normalized) # Alberta COVID-19 active case rate per 100,000 (normalized)	517.1 (34.7, 7713.4)	0.000	N/A	N/A
Time in state (mask mandate on or off)	0.984 (0.978, 0.990)	0.000	N/A	N/A
Government mandatory masking # Time in state	1.01 (1.005, 1.02)	0.004	N/A	N/A
Government mandatory masking # Alberta COVID-19 active case rate per 100,00 (nor) # Time in state	1.04 (1.02, 1.07)	0.000	N/A	N/A

© 2023 Hahn LM et al. *JAMA Network Open.*

eTable 5: Child avoiding crowded places/gatherings (often or always): Supplementary analysis

	Child avoiding crowded places/gatherings (parent report)			
	Odds ratio (95%CI)	p-value	Risk ratio (95%CI)	p-value
Parent vaccine: not willing to receive and not vaccinated	0.66 (0.48, 0.93)	0.02	1.1 (0.98, 1.2)	0.12
Parent vaccine: not available and willing to receive	0.91 (0.68, 1.2)	0.54	1.0 (0.96, 1.1)	0.82
Parent vaccine: missing	0.62 (0.40, 0.96)	0.03	0.93 (0.85, 1.0)	0.12
Child mask use (often or always)	3.0 (2.4, 3.8)	0.000	1.3 (1.2, 1.4)	0.000
Indoor restrictions (limit of 15 or 10 people)	0.94 (0.81, 1.1)	0.45	0.95 (0.93, 0.97)	0.000
Indoor restrictions (no gatherings)	2.0 (1.6, 2.6)	0.000	1.1 (1.1, 1.2)	0.000
Alberta COVID-19 active case rate per 100,000 (normalized)	1.4 (1.1, 1.9)	0.01	N/A	N/A
Location: Rural (outside of Edmonton or Calgary)	0.56 (0.44, 0.71)	0.000	0.90 (0.85, 0.94)	0.000
Time throughout the study	0.99 (0.99, 0.96)	0.000	N/A	N/A

© 2023 Hahn LM et al. *JAMA Network Open.*

eTable 6: A comparison of demographics for participants who withdrew from the study and participants who stayed in the study

	Participants who **withdrew** from the study (53/939) n (mean)	Participants who **stayed in the study** (886/939) n (mean)	p-value
Age of child at baseline	53/53 (10.49)	886/886 (10.61)	0.59
Sex of child at birth (female, male)	24/52 (46.2), 28/52 (53.8)	433/877 (49.4), 443/877 (50.5)	0.80
Household income at baseline	49/53 (110857)	873/886 (112617.8)	0.66
Home in rural location	19/51 (37.3)	305/884 (34.5)	0.69
Parent education: High school or higher	45/51 (88.2)	822/879 (93.5)	0.15
Parent education: University Bachelor's degree or higher	28/51 (54.9)	564/879 (64.2)	0.18
Parent working in a hospital or a health care facility worker	11/53 (20.8)	244/886 (27.5)	0.28
Parent a first responder (paramedic, firefighter, police officer)	3/53 (5.7)	39/886 (4.4)	0.67
Parent a childcare worker	1/53 (1.9)	22/886 (2.5)	0.79
Parent a teacher	8/53 (15.1)	94/886 (10.6)	0.31

© 2023 Hahn LM et al. *JAMA Network Open.*

eTable 7: Child mask use (often or always) with missing data incorporated into the largest category

	Missing as a separate category		Missing incorporated into largest category	
	Odds ratio (95%CI)	p-value	Odds ratio (95%CI)	p-value
Government mandatory masking	18.3 (5.7, 58.6)	0.000	21.9 (6.6, 73.0)	0.000
Parent vaccine: not willing to receive and not vaccinated	0.49 (0.29, 0.81)	0.006	0.47 (0.27, 0.78)	0.004
Parent vaccine: not available and willing to receive	1.2 (0.52, 2.8)	0.66	1.2 (0.50, 2.7)	0.74
Avoided crowded places/gatherings (often or always)	5.4 (4.2, 7.0)	0.000	5.7 (4.5, 7.4)	0.000
Alberta COVID-19 active case rate per 100,000 (normalized)	0.02 (0.05, 0.1)	0.000	0.01 (0.003, 0.06)	0.000
Alberta COVID-19 active case rate per 100,000 (normalized) # Time in state	1.00 (0.99, 1.01)	0.80	1.0 (0.98, 1.01)	0.56
Government mandatory masking # Alberta COVID-19 active case rate per 100,000 (normalized)	0.0005 (0.00003, 0.009)	0.000	0.0004 (0.00003, 0.008)	0.000
Alberta COVID-19 active case rate per 100,000 (normalized) # Alberta COVID-19 active case rate per 100,000 (normalized)	517.1 (34.7, 7713.4)	0.000	1381.48 (95.5, 19973.6)	0.000
Time in state (mask mandate on or off)	0.984 (0.978, 0.990)	0.000	0.988 (0.982, 0.993)	0.000
Government mandatory masking # Time in state	1.01 (1.005, 1.02)	0.004	1.01 (1.001, 1.02)	0.03
Government mandatory masking # Alberta COVID-19 active case rate per 100,00 (nor) # Time in state	1.04 (1.02, 1.07)	0.000	1.05 (1.02, 1.07)	0.000

© 2023 Hahn LM et al. *JAMA Network Open.*

eTable 8: Child mask use and avoiding crowded places/gatherings: Trajectory group analysis

	Child mask use (parent report)			Child avoiding crowded places/gathering (parent report)		
	Group 1 *Lower* OR (p-value) (95%CI)	Group 2 *Intermediate* *Reference group	Group 3 *Higher* OR (p-value) (95%CI)	Group 1 *Lower* OR (p-value) (95%CI)	Group 2 *Intermediate* *Reference group	Group 3 *Higher* OR (p-value) (95%CI)
Income (2nd quartile)	1.1 (0.04) (1.0, 1.2)	*	0.76 (0.000) (0.70, 0.82)	0.75 (0.000) (0.66, 0.86)	*	0.59 (0.000) (0.56, 0.63)
Income (3rd quartile)	0.90 (0.01) (0.82, 1.0)	*	0.69 (0.000) (0.64, 0.74)	1.2 (0.000) (1.1, 1.4)	*	0.70 (0.000) (0.65. 0.75)
Income (4th quartile)	0.82 (0.000) (0.76, 0.89)	*	0.50 (0.000) (0.47, 0.54)	1.1 (0.06) (1.0, 1.3)	*	0.69 (0.000) (0.64, 0.74)
Parent education: High school	1.4 (0.31) (0.75, 2.5)	*	2.2 (0.20) (1.1, 4.1)	0.57 (0.16) (0.27, 1.2)	*	1.2 (0.58) (0.65, 2.1)
Parent: education: University	1.0 (0.96) (0.71, 1.4)	*	1.0 (0.79) (0.76, 1.4)	0.81 (0.38) (0.50, 1.3)	*	1.2 (0.34) (0.86, 1.6)
Parent occupation: Work in hospital or health care facility worker	1.0 (0.29) (1.0, 1.1)	*	0.95 (0.11) (0.89, 1.0)	0.98 (0.64) (0.89, 1.1)	*	0.70 (0.000) (0.66, 0.74)
Parent vaccine willingness	0.55 (0.000) (0.40, 0.75)	*	2.0 (0.000) (1.4, 2.9)	0.14 (0.000) (0.09, 0.02)	*	1.4 (0.08) (0.97, 1.9)
Location: Rural (outside of Edmonton or Calgary)	0.94 (0.28) (0.83, 1.1)	*	0.57 (0.000) (0.51, 0.64)	2.2 (0.000) (1.8, 2.6)	*	0.70 (0.000) (0.63, 0.78)
Location: Urban (Edmonton or Calgary)	1.1 (0.28) (0.95, 1.2)	*	1.8 (0.000) (1.6, 2.0)	0.46 (0.000) (0.39, 0.54)	*	1.4 (0.000) (1.3, 1.6)

© 2023 Hahn LM et al. *JAMA Network Open.*

eTable 9. Chid avoiding crowded places/gatherings (often or always) with missing data incorporated into the largest category

	Missing as a separate category		Missing incorporated into largest category	
	Odds ratio (95%CI)	p-value	Odds ratio (95%CI)	p-value
Parent vaccine: not willing to receive and not vaccinated	0.66 (0.48, 0.93)	0.02	0.72 (0.52, 1.0)	0.06
Parent vaccine: not available and willing to receive	0.91 (0.68, 1.2)	0.54	0.98 (0.74, 1.3)	0.87
Child mask use (often or always)	3.0 (2.4, 3.8)	0.000	3.0 (2.4, 3.8)	0.000
Indoor restrictions (limit of 15 or 10 people)	0.94 (0.81, 1.1)	0.45	0.95 (0.82, 1.1)	0.49
Indoor restrictions (no gatherings)	2.0 (1.6, 2.6)	0.000	2.2 (1.7, 2.8)	0.000
Alberta COVID-19 active case rate per 100,000 (normalized)	1.4 (1.1, 1.9)	0.01	1.2 (0.94, 1.6)	0.13
Location: Rural (outside of Edmonton or Calgary)	0.56 (0.44, 0.71)	0.000	0.56 (0.45, 0.71)	0.000
Time throughout the study	0.99 (0.99, 0.96)	0.000	1.0 (0.99, 1.0)	0.000

© 2023 Hahn LM et al. *JAMA Network Open.*

eFigure 1: Participant recruitment and sample size

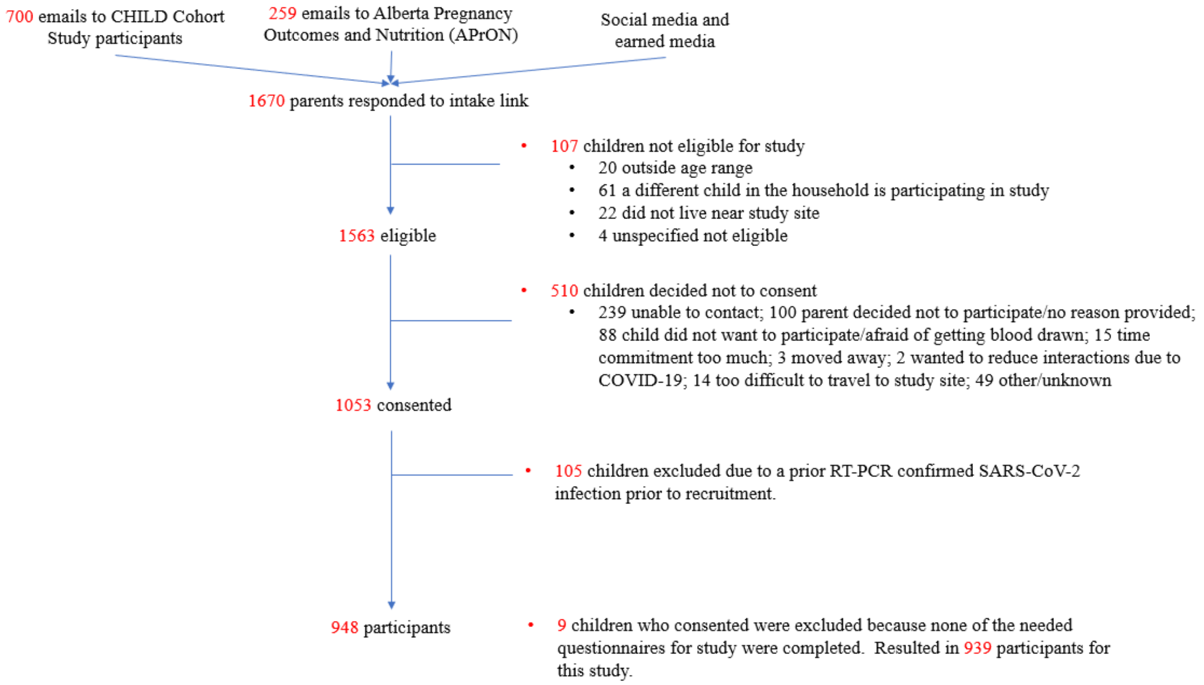

© 2023 Hahn LM et al. *JAMA Network Open.*

eFigure 2: Participant response throughout the study

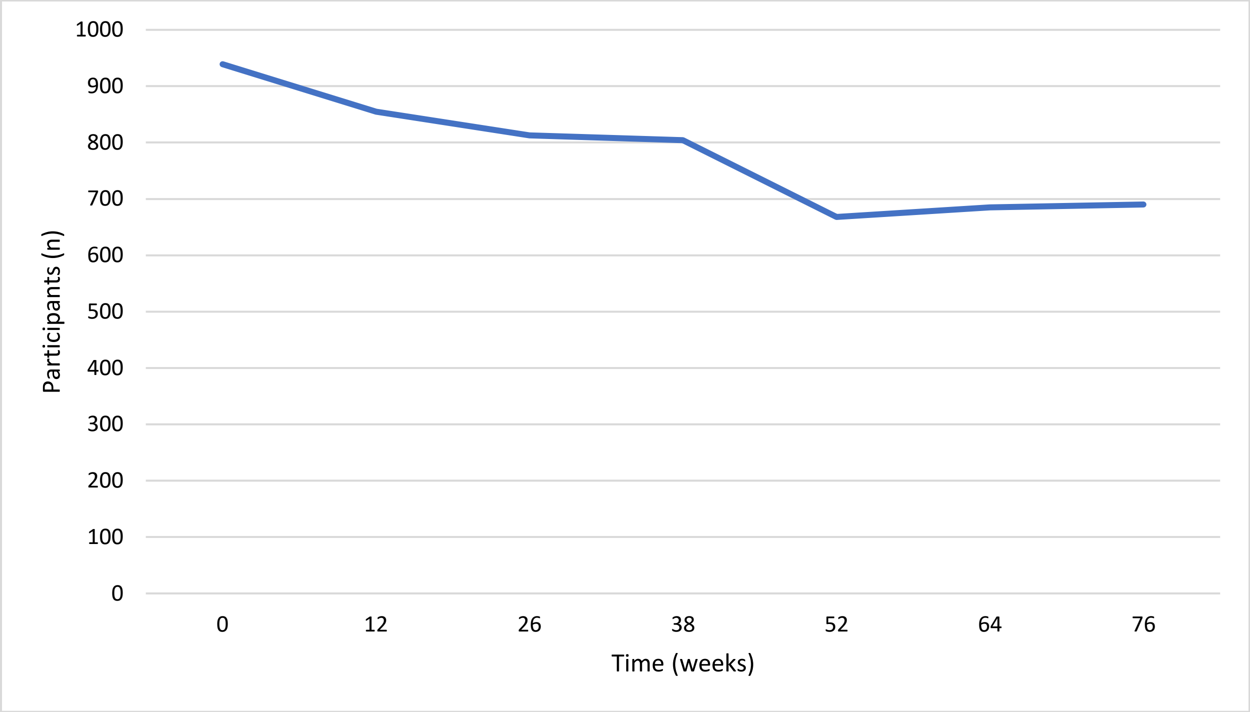

© 2023 Hahn LM et al. *JAMA Network Open.*

eFigure 3: Child mask use (percentage)

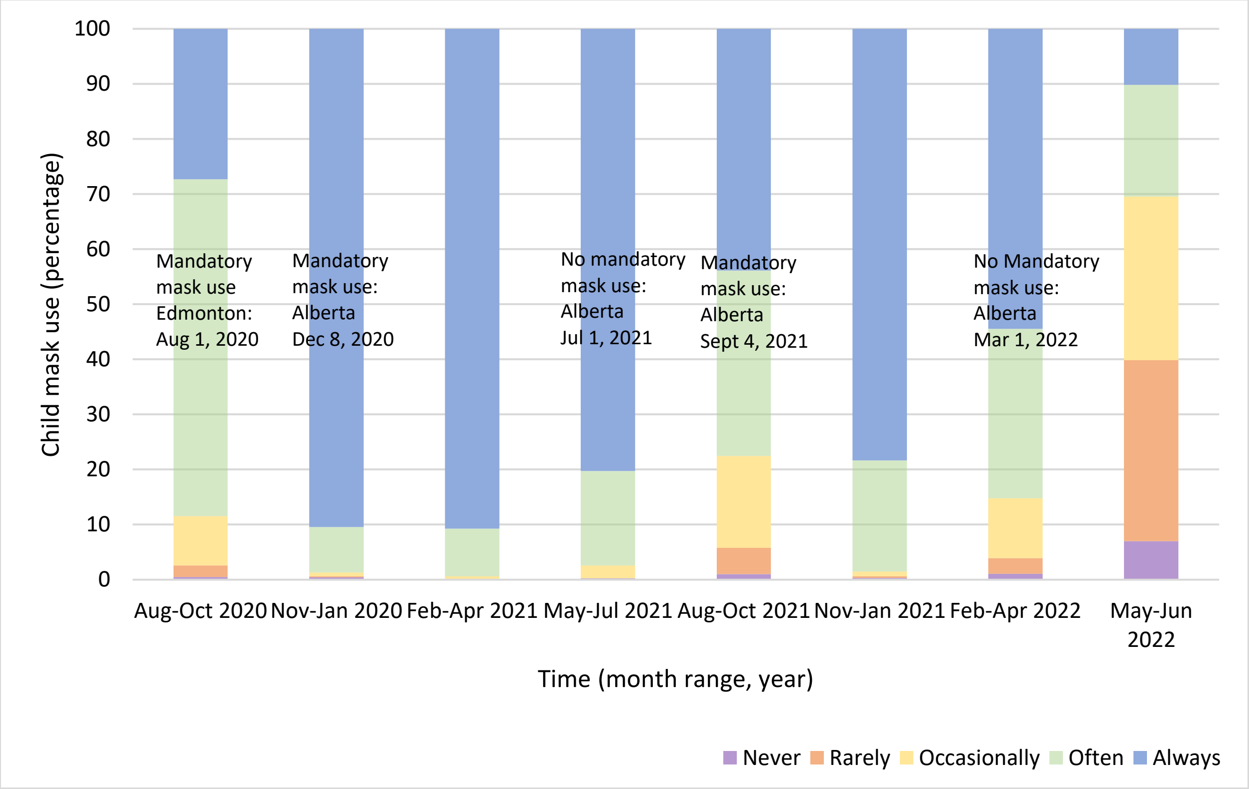

© 2023 Hahn LM et al. *JAMA Network Open.*
