# Supplementary material for: Association of COVID-19 Government-Instituted Mask Mandates With Incidence of Mask Use Among Children in Alberta, Canada
Source: JAMA Netw Open. 2023 Jun 8;6(6):e2317358. doi: 10.1001/jamanetworkopen.2023.17358 (PMC10251214; doi:10.1001/jamanetworkopen.2023.17358)
Supplement: Supplement 2. — Data Sharing Statement [file jamanetwopen-e2317358-s002.pdf]

Data Sharing Statement

Hahn. Association of COVID-19 Government-Instituted Mask Mandates With Incidence of Mask Use Among Children in Alberta, Canada. *JAMA Netw Open*. Published June 08, 2023. doi:10.1001/jamanetworkopen.2023.17358

Data
Data available: No

Additional Information
Explanation for why data not available: Participant data. We will work with interested groups on sharing data
